# Supplementary material for: Acetate Alleviates Gut Microbiota Depletion-Induced Retardation of Skeletal Muscle Growth and Development in Young Mice
Source: Int J Mol Sci. 2024 May 8;25(10):5129. doi: 10.3390/ijms25105129 (PMC11121558; doi:10.3390/ijms25105129)
Supplement: Supplementary file 1 [file ijms-25-05129-s001.zip › Supplementary Table and Figures of this study.pdf]

Supplementary Table and Figures of “Acetate alleviates gut microbiota depletion-induced retardation of skeletal muscle growth and development in young mice”

Gene	Accession No.	Primer sequence (5' to 3')
Myod1	NM_010866.2	F: TGCTCTGATGGCATGATGGAT
		R: AGATGCGCTCCACTATGCTG
Myog	NM_031189.2	F: GTCCCAACCCAGGAGATCATT
		R: AGTTGGGCATGGTTTCGTCT
Myf6	NM_002469.3	F: GTGGACCCCTACAGCTACAA
		R: ACGTTTGCTCCTCCTTCCTTA
Mef2a	NM_001033713.2	F: GTTCACTCGTGTCACCGTCT
		R: GCTCAACATCCCACTTGCAC
ACTB (reference)	NM_007393.5	F: CTTTTCCAGCCTTCCTTCTTG
		R: TTGGCATAGAGGTCTTTACGG
Gm16062	NR_045686.1	F: TGGAACCCGCATGGTACTTC
		R: TTCTCCTTCACAGAGCATGGG
miR-129-2-3p	LM379112.1	F: AAGCCCTTACCCCAAAAAGCAT

Table S1. Primer sequences for RT-qPCR

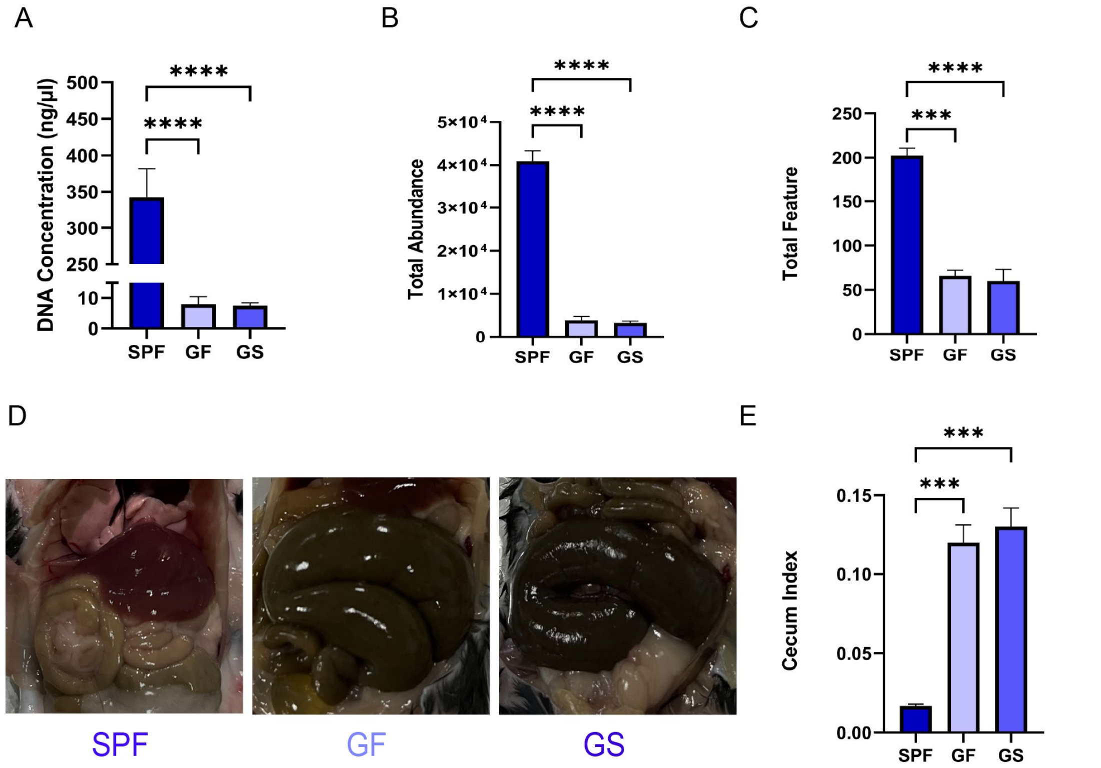

Figure S1. Detection of the microbial content of the mice among the groups. A. Total fecal microbial DNA concentration detected by spectrophotometry. B. The total fecal microbial abundance analyzed by QIIME2. C. The number of fecal microbial species analyzed by QIIME2. D. Cecal morphology of mice. F. The cecal index calculated by dividing cecal weight by body weight.. All data are expressed as the mean ± SEM (n = 3 per group) and the "*n*" defines the number of biological replicates. Data were analyzed using one-way ANOVA test and were considered statistically significant, at ***P < 0.001 and ****P < 0.0001 between the indicated groups.

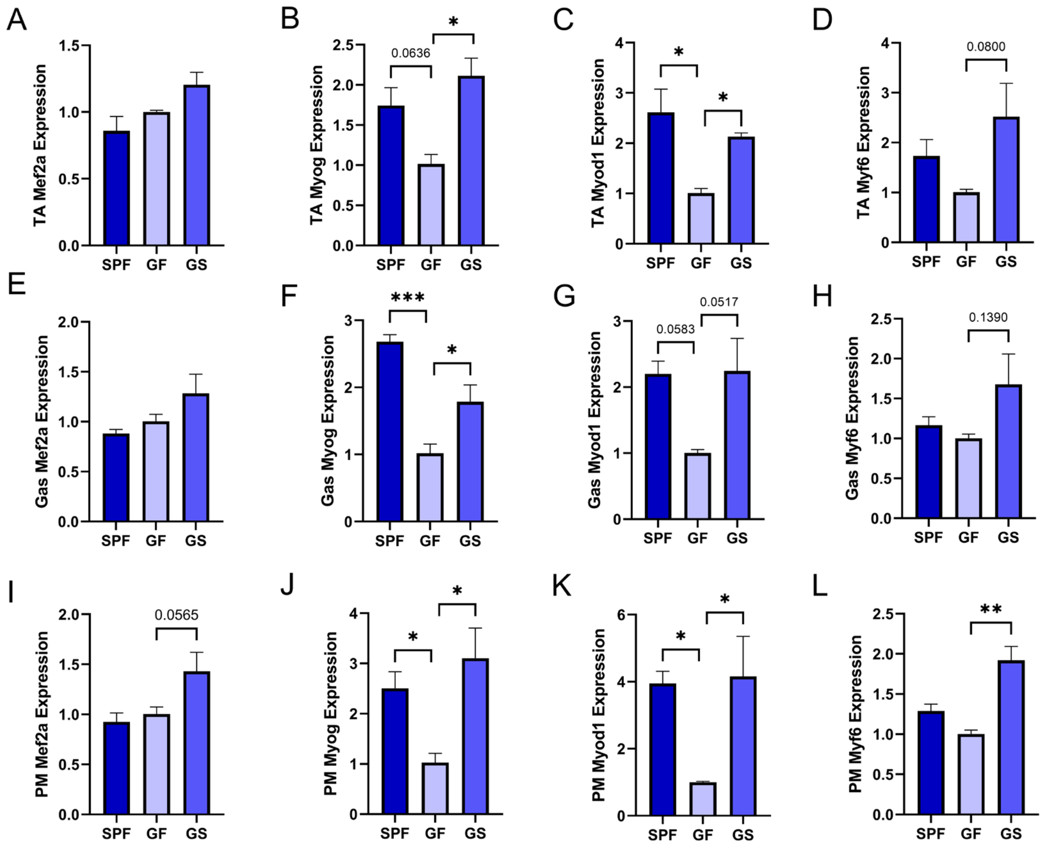

Figure S2. Acetate relieved the gut microbiota depletion-induced inhibition of the MRFs expression in skeletal muscle. A-L. Detection of *Mef2a*, *Myod1*, *Myog* and *Myf6* expression in TA (A-D), Gas (E-H) and PM muscle (I-L) using RT-qPCR, respectively. All data are expressed as the mean ± SEM (n = 3 per group), and the "*n*" defines the number of biological replicates. Data were analyzed using one-way ANOVA test and were considered statistically significant, at *P < 0.05, **P < 0.01 , and ***P < 0.01 between the indicated groups.

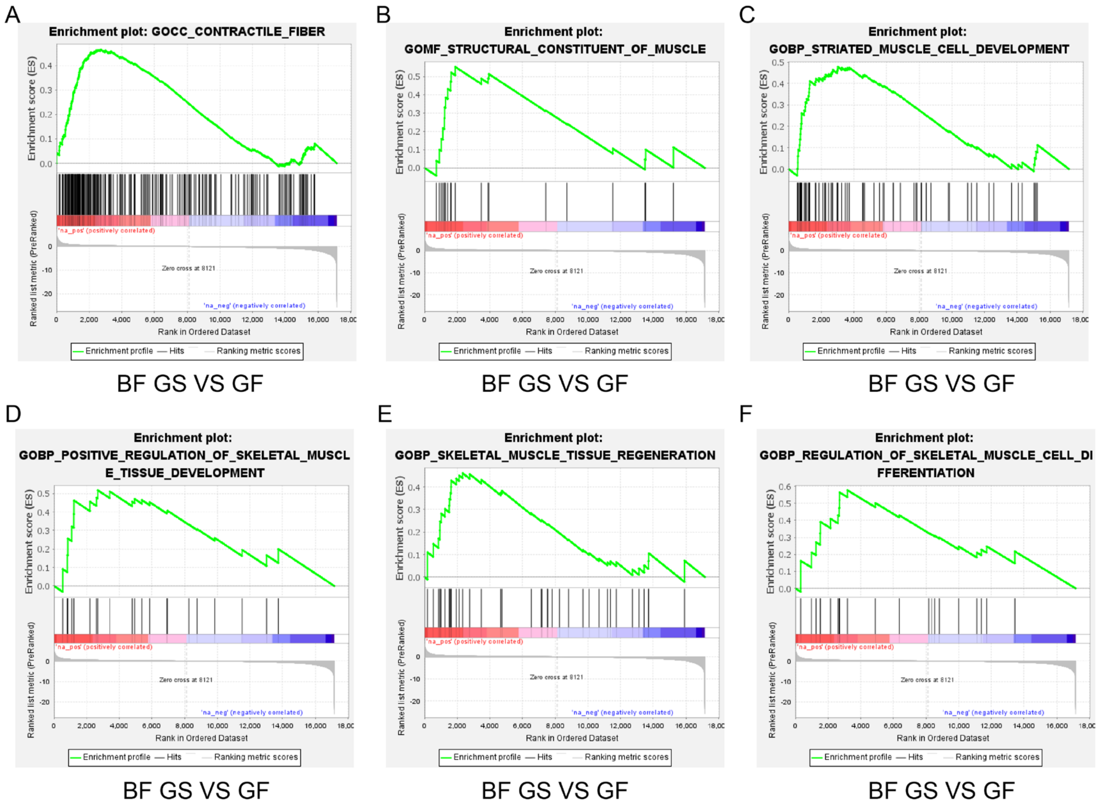

Figure S3. GSEA revealed that acetate promoted the growth and development of BF in the GS group. A-F. Compared to GF group, acetate promoted the "contractile fiber" (A), "structural constituent of muscle" (B), "striated muscle cell development" (C), "positive regulation of skeletal muscle tissue development" (D), "skeletal muscle tissue regeneration" (E), "regulation of skeletal muscle cell differentiation" (F) biological process in the BF of the GS group. A permutation test was applied to the analysis. n = 3 per group and the "*n*" defines the number of biological replicates.

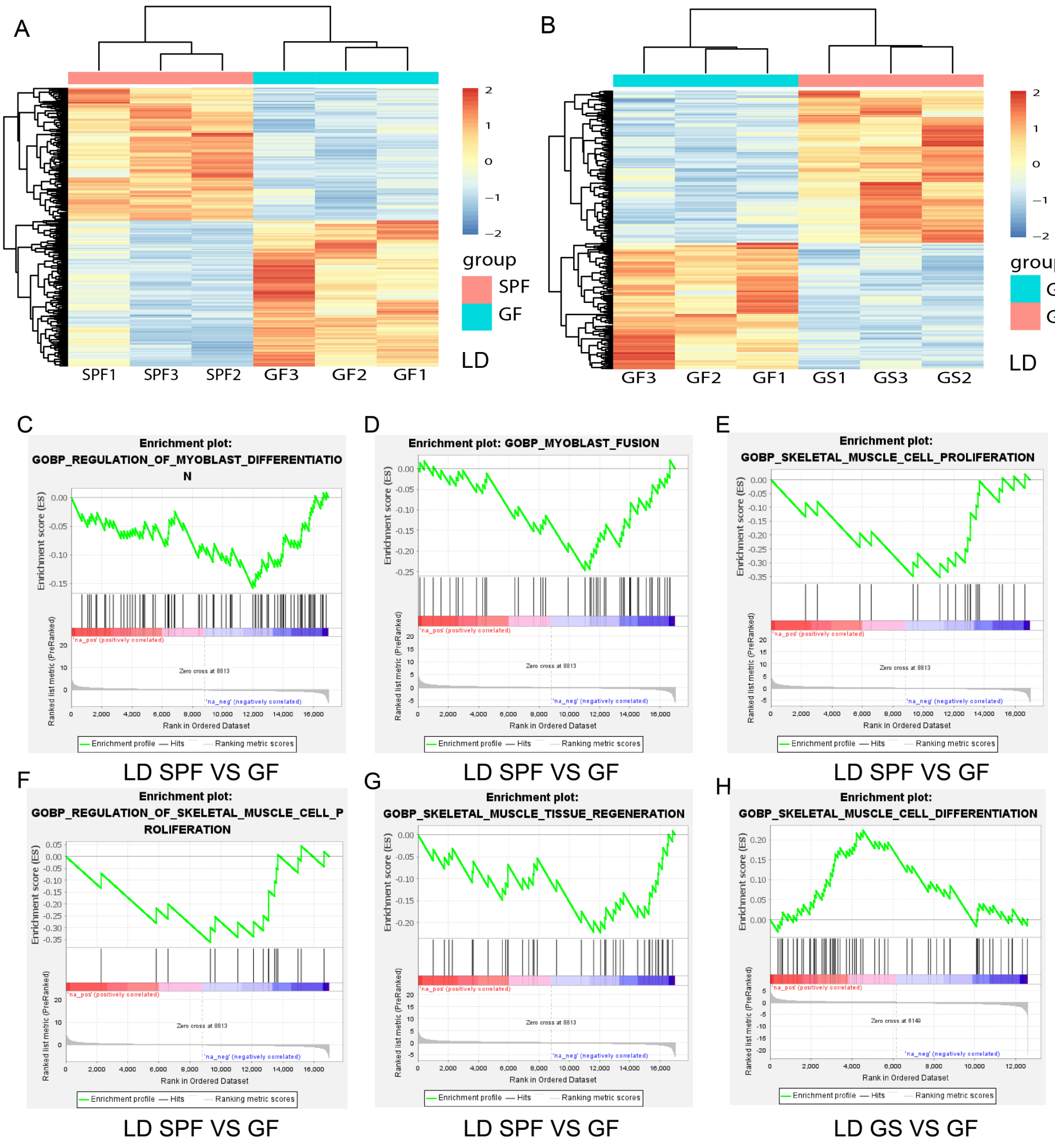

Figure S4. Transcriptome differences among groups of LD and GSEA revealed that acetate alleviated the growth and development inhibition of LD in mice induced by the absence of gut microbiota. A, B. Heatmap of the differentially expressed genes in the SPF *vs.* GF (A) and GF *vs.* GS (B). C-G. Compared to SPF group, loss of gut microbiota inhibited "regulation of myoblast differentiation" (C), "myoblast fusion" (D), "skeletal muscle cell proliferation" (E, F), "skeletal muscle tissue regeneration" (G) biological process in the LD muscle of the GF group. H. Compared to GF group, acetate promoted the biological process of "skeletal muscle cell differentiation" biological process in the LD of the GS group. A permutation test was applied to the analysis. n = 3 per group and the "*n*" defines the number of biological replicates.

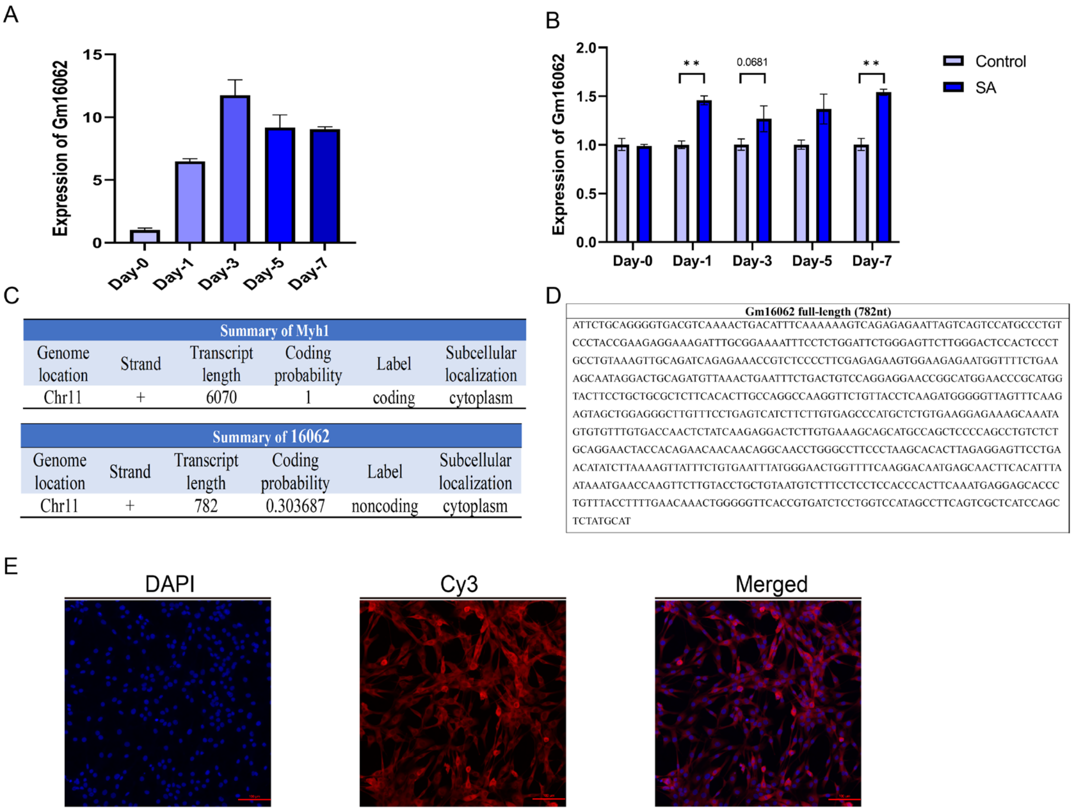

Summary of Myh1					
Genome location	Strand	Transcript length	Coding probability	Label	Subcellular localization
Chr11	+	6070	1	coding	cytoplasm

Summary of 16062					
Genome location	Strand	Transcript length	Coding probability	Label	Subcellular localization
Chr11	+	782	0.303687	noncoding	cytoplasm

Gm16062 full-length (782nt)

ATTCTGCAGGGGTGACGTCAAAACTGACATTTCAAAAAAGTCAGAGAGAATTAGTCAGTCCATGCCCTGT
CCCTACCGAAGAGGAAAGATTTGCGGAAAATTTCCTCTGGATTCTGGGAGTTCTTGGGACTCCACTCCCT
GCCTGTAAAGTTGCAGATCAGAGAAACCGTCTCCCCTTCGAGAGAAGTGGAAGAGAATGGTTTTCTGAA
AGCAATAGGACTGCAGATGTTAAACTGAATTTCTGACTGTCCAGGAGGAACCGGCATGGAACCCGCATGG
TACTTCCTGCTGCGCTCTTCACACTTGCCAGGCCAAGGTTCTGTTACCTCAAGATGGGGGTTAGTTTCAAG
AGTAGCTGGAGGGCTTGTTTCCTGAGTCATCTTCTTGTGAGCCCATGCTCTGTGAAGGAGAAAGCAAATA
GTGTGTTTGTGACCAACTCTATCAAGAGGACTCTTGTGAAAGCAGCATGCCAGCTCCCCAGCCTGTCTCT
GCAGGAACTACCACAGAACAACAACAGGCAACCTGGGCCTTCCCTAAGCACACTTAGAGGAGTTCCTGA
ACATATCTTAAAAGTTATTTCTGTGAATTTATGGGAACTGGTTTTCAAGGACAATGAGCAACTTCACATTTA
ATAAATGAACCAAGTTCTTGTACCTGCTGTAATGTCTTTCCTCCTCCACCCACTTCAAATGAGGAGCACCC
TGTTTACCTTTTGAACAAACTGGGGGTTCACCGTGATCTCCTGGTCCATAGCCTTCAGTCGCTCATCCAGC
TCTATGCAT

Figure S5 Expression patterns and characteristics of *Gm16062*. A. *Gm16062* expression during C2C12 myogenic differentiation was determined using RT-qPCR with 2mmol/L SA supplement (n = 3 per group). B. *Gm16062* expression during C2C12 differentiation between control group and SA supplementation group was detected using RT-qPCR. C. The protein coding ability and subcellular localization of *Gm16062* predicted by Annolnc2. D. The full-length transcript sequence of *Gm16062*. E. On 3rd day of C2C12 myogenic differentiation, the subcellular environment of *Gm16062* was localized with a Cy3-labeled oligonucleotide probe (20×, scale bar, 100 μm). All data are expressed as the mean ± SEM (n = 3 per group) and the "*n*" defines the number of biological replicates. Data were analyzed using an unpaired two tailed Student's *t* test and were considered statistically significant, at $^{**}P < 0.01$ between the indicated groups.

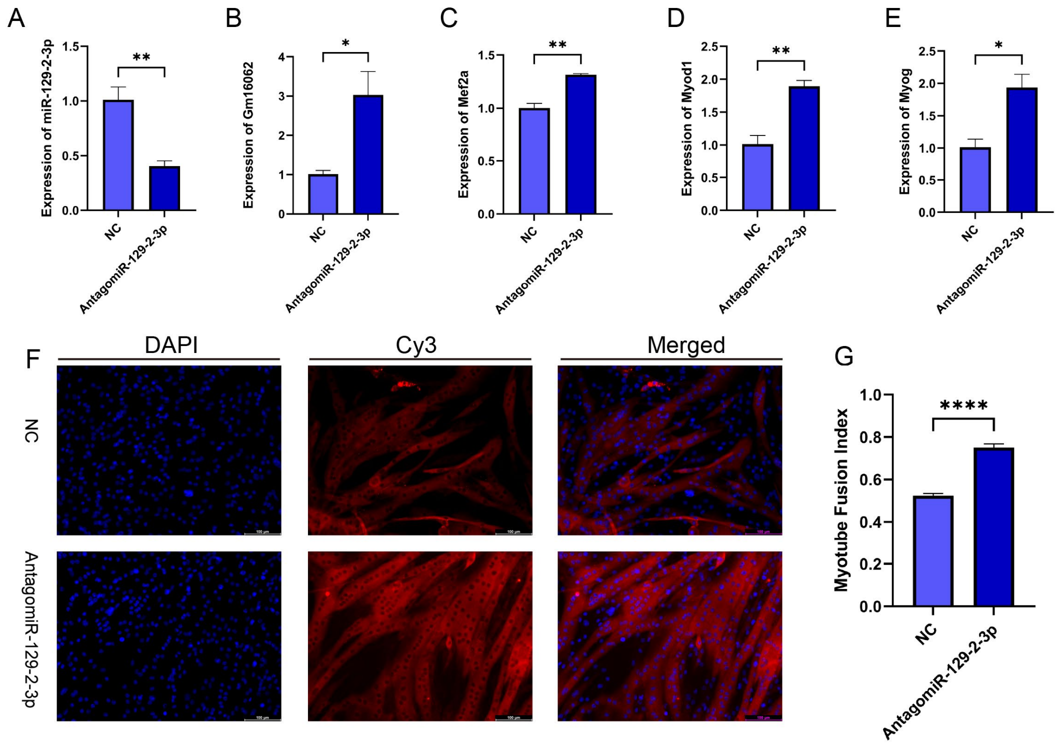

Figure S6. Knockdown of *miR-129-2-3p* expression promoted C2C12 myogenic differentiation. A. The transfection efficiency of antagomiR-129-2-3p 48 h after transfection was detected using RT-qPCR (n = 3 per group). B-E. The expression levels of *Gm16062* (B), *Mef2a* (C), *Myod1* (D), and *Myog* (E) were detected using RT-qPCR on the 5th day of C2C12 differentiation transfected with antagomiR-129-2-3p (n = 3 per group). F. MYH4 was detected using immunofluorescence staining on the 5th day of C2C12 differentiation transfected with antagomiR-129-2-3p (20×, scale bar, 100 μm; n = 5 per section per group). G. The myotube fusion index was calculated using ImageJ (n = 5 per group). All data are expressed as the mean ± SEM and the "*n*" defines the number of biological replicates. Data were analyzed using an unpaired two tailed Student's *t* test and were considered statistically significant, at *P < 0.05, **P < 0.01, and ****P < 0.001 between the indicated groups.
